# Near Infrared Emitting Semiconductor Polymer Dots for Bioimaging and Sensing

**DOI:** 10.3390/s22197218

**Published:** 2022-09-23

**Authors:** Connor Riahin, Kushani Mendis, Brandon Busick, Marcin Ptaszek, Mengran Yang, Gary Stacey, Amar Parvate, James E. Evans, Jeremiah Traeger, Dehong Hu, Galya Orr, Zeev Rosenzweig

**Affiliations:** 1 Department of Chemistry and Biochemistry, University of Maryland Baltimore County, Baltimore, MD 21250, USA; 2 Divisions of Plant Sciences and Biochemistry, University of Missouri, Columbia, MO 65211, USA; 3 Environmental Molecular Sciences Laboratory (EMSL), Pacific Northwest National Laboratory, Richland, WA 99354, USA; 4 School of Biological Sciences, Washington State University, Pullman, WA 99164, USA

**Keywords:** near infrared emission, polymer dots, plant cell imaging, sensing

## Abstract

Semiconducting polymer dots (Pdots) are rapidly becoming one of the most studied nanoparticles in fluorescence bioimaging and sensing. Their small size, high brightness, and resistance to photobleaching make them one of the most attractive fluorophores for fluorescence imaging and sensing applications. This paper highlights our recent advances in fluorescence bioimaging and sensing with nanoscale luminescent Pdots, specifically the use of organic dyes as dopant molecules to modify the optical properties of Pdots to enable deep red and near infrared fluorescence bioimaging applications and to impart sensitivity of dye doped Pdots towards selected analytes. Building on our earlier work, we report the formation of secondary antibody-conjugated Pdots and provide Cryo-TEM evidence for their formation. We demonstrate the selective targeting of the antibody-conjugated Pdots to FLAG-tagged FLS2 membrane receptors in genetically engineered plant leaf cells. We also report the formation of a new class of luminescent Pdots with emission wavelengths of around 1000 nm. Finally, we demonstrate the formation and utility of oxygen sensing Pdots in aqueous media.

## 1. Introduction

**Luminescent semiconducting polymer dots**—Semiconductor polymer dots (Pdots) have rapidly garnered interest as an alternative to semiconductor quantum dots and fluorescent dyes in nanoscale fluorescence imaging and sensing applications [1]. Pdots are nanoparticles ranging from 10 to 100 nm in diameter. They are primarily composed of a semiconducting polymer and additives to enable their aqueous solubility and unique emission properties [2]. When synthesized with no dopant dyes, the formed Pdots largely retain the optical properties of the bulk semiconducting polymer, with decreased quantum yield and the appearance of a red emission tail being the only noticeable differences. Luminescent Pdots are characterized with low batch-to-batch variance in emission properties. This is in contrast to the high variance in emission properties often observed with other semiconductor luminescent nanoparticles like inorganic quantum dots. Pdots have several desirable properties that make them attractive alternatives to other luminescent nanoparticles. The high brightness of Pdots is owed to their fast emissive rates and large extinction coefficients [3]. Since each monomer with the polymer chain is capable of absorbing incident light, the high concentration of chromophores in Pdots gives them extinction coefficients an order of magnitude higher than luminescent quantum dots and two orders of magnitude greater than organic dyes [4]. Furthermore, Pdots are highly resistant to photobleaching, which make them excellent candidates for fluorescence imaging and sensing applications [1]. Recent advancements in polymer design have produced drastic enhancements in quantum yield, up to 0.98 [5]. Finally, Pdots exhibit minimal or no cytotoxicity towards mammalian cells which distinguish them from heavy metal, cadmium and lead containing quantum dots [6].

**Scheme 1:**
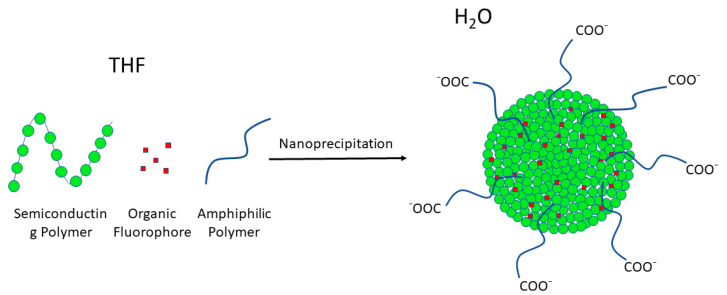
Nanoprecipitation of a semiconducting polymer, for example Poly(fluorine-alt-benzothiadiazole) (PFBT), an organic fluorophore, for example phthalocyanine, and an amphiphilic polymer, for example polystyrene maleic anhydride (PSMA), all dissolved in tetrahydrofuran (THF), to form highly emitting polymer dots when injected into aqueous solution.

**Polymer Dot Synthesis**—One of the greatest advantages of Pdots is their low-cost, facile synthesis. Although there are several methods by which to prepare Pdots, the one that produces the smallest Pdots and is most commonly used is the nanoprecipitation method [7]. A schematic diagram which describes the nanoprecipitation used in our laboratory and others to form near infrared highly emitting dye-doped Pdots is shown in Scheme 1. In this method, the precursor materials including the semiconducting polymer, a secondary amphiphilic polymer which provides carboxyl groups for Pdots solubility in aqueous solution, and the doped dye (if used) are dissolved in a water-miscible organic solvent like tetrahydrofuran (THF). A sample of this solution is rapidly injected into a larger volume of water under vigorous sonication. The hydrophobic nature of the semiconducting polymers drives their aggregation into spherical particles to minimize hydrophobic interactions. The THF is then removed, and the particles filtered to remove any large aggregates. The process, from precursors to purified Pdots, can take as little as 10 min and requires no specialized equipment, making Pdots one of the simplest nanoparticles to produce. In addition to the semiconducting polymer, additional components can be mixed into the Pdots to alter their surface and optical properties. In most cases, an amphiphilic surfactant is included to increase stability and provide binding sites for future functionalization. This is most commonly a poly(styrene) co-polymer with carboxylated poly (ethylene glycol) or maleic anhydride, though the use of electrolytes [8], lipids [9], and dextran [10] have also been reported. The hydrophobic end of the surfactant is incorporated into the polymer matrix during the formation of the Pdots, while the hydrophilic end remains on the surface, providing electrostatic and steric repulsion to reduce aggregation, as well as functional groups for further functionalization. 

Earlier studies have shown that the optical properties of Pdots can be tuned through the use of a dopant fluorescent dye [11]. For example, the inclusion of fluorescent dyes in Pdot synthesis results in the dye being embedded in the polymer matrix. Partial overlap between the polymer emission and dye absorption provides the conditions for efficient energy transfer between the polymer and the dye. The resultant Pdots show significantly narrowed and red-shifted emission compared to the non-doped Pdots. The dopant can either be mixed with the precursors during the synthesis or covalently bound directly to the semiconducting polymer itself [12]. The covalent binding method has been reported to impart greater photostability to the Pdots at the cost of the additional complexity of synthesizing the polymer.

**Polymer dots in Bioimaging applications**—Several studies have investigated the application of various Pdots as nanoscale bioimaging probes. Pdots interactions and cell permeation have been demonstrated [13,14]. Due to their powerful optical properties, Pdots have been extensively investigated as fluorescence probes for fluorescence imaging. For specific cellular imaging, the surface of Pdots is modified by coupling the carboxyl groups on the surface of the Pdots to a cellular targeting moiety, for example an antibody towards a cellular target, often using an *N*-(3-Dimethylaminopropyl)-*N*′-ethylcarbodiimide (EDC) coupling reaction. Many studies make use of streptavidin to target biotinylated antibodies [15,16], though the use of IgG secondary antibodies [17] and tetrameric antibody complexes [10] has also been reported. The high per-particle brightness, resistance to photobleaching, selective imaging capabilities of Pdots-antibody conjugates, and the inherent photoblinking properties of Pdots make them ideal candidates for wide field, confocal and super-resolution cellular imaging [18,19]. As of now, the highest reported resolution using Pdots is 57 nm, several times smaller than the diffraction limit of light [16].

**Polymer Dots as Fluorescence Sensors**—Green emitting Pdots were used for the detection of alkaline phosphatase (ALP) activity [20], and to detect dopamine in living cells [21]. pH sensing Pdots were formed by coupling the pH sensitive dye fluorescein and squarine dyes to Pdots [22,23]. These pH sensing Pdots feature ratiometric sensing capabilities and linear range within biologically relevant pH levels between pH 5 and 8. Recently a dual-function Pdot for pH sensing and singlet oxygen generation for photodynamic therapy (PDT) was also developed [24,25]. Ratiometric and fluorescence lifetime–based ion sensing Pdots were developed, for example to measure fluoride levels in water and biological media [26]. These Pdots were able to accurately sense F- within relevant concentration in both aqueous media and living cells. A unique strategy for lead sensing was developed based on purposeful dye leakage from Pdots [27]. Pdot-based oxygen sensors have been developed by doping the Pdots with platinum (II) porphyrin, an oxygen sensitive dye [28]. A similar Pt-Pdot in which made use of two-photon excitation was also developed [29]. The Pt-Pdots were excited at 740 nm and produced a high signal to noise ratio when used in cells. Oxygen sensing Pdots were also formed by doping the Pdots with an iridium (III) complex, which was used both as an oxygen sensitive dye and as a sensitizer for photo dynamic therapy [30]. The oxygen sensing properties of Pdots doped with transition metal complexes and glucose oxidase were used to form a wireless glucose sensor that could be used with a smartphone [31]. 

Recently we reported the synthesis of porphyrin (chlorin and bacteriochlorin)-based Pdots and their use for cellular imaging in plants [32]. NIR emitting Pdots are necessary for plant cell imaging because of optical interference by chlorophyll and lignin. In our previous work we demonstrated the multiplexing capabilities of luminescent Pdots, which can be excited by a single wavelength light source but tunable emission peak wavelength. We also showed that the resulting Pdots have no measurable cytotoxicity. In this paper, which builds on our previous work, we describe the formation of Pdots with the longest emission wavelength to date. We demonstrate for the first time the use of Cryo-TEM to confirm the formation of antibody-coated Pdots and demonstrate their use in targeting FLAG-tagged FLS2 membrane receptors in genetically engineered plant leaf cells. Expanding Pdots imaging and sensing capabilities to the near infrared is imperative to successfully image or sense molecular targets in complex biological matrices that show high levels of autofluorescence and light scattering. In such matrices, for example plant cells and tissue, the ability to image molecular targets in the visible region of the electromagnetic spectrum is greatly impaired Cellular and tissue autofluorescence and light scattering present minimal or no interference with fluorescence signals of near infrared emitting Pdots despite the use of a visible excitation wavelength. 

## 2. Experimental Methods

**Chemical Reagents**—Poly[(9,9-dioctylfluorenyl-2,7-diyl)-alt-co-(1,4-benzo-(2,1′,3)-thiadiazole)] (PFBT) (37,000 MW, 3.3 polydispersity) and Poly [2-(5-cyano-methylhexyloxy)-1-4-phenylene] (CNPPP) (40,000 MW, 3.0 polydispersity) were purchased from American Dye Source, Baie-D’Urfe, QC, Canada. Poly (styrene/maleic anhydride) (PSMA) [67:33] (MW 7500) was purchased from Polysciences, Warrington, PA, USA. Palladium catalysts and thionyl chloride were obtained from Sigma-Aldrich, St. Louis, MO, USA. Diamine and boronic acids were obtained from Ambeed, Arlington Heights, IL, USA. Tetrahydrofuran (anhydrous, > 99.8%), Phosphate Buffered Saline (PBS), and Bovine Serum Albumin (BSA) were purchased from Fisher Scientific, Hampton, NH, USA. Dulbecco’s Modified Eagle Medium, Fetal Bovine Serum, HEPES buffer and Sodium Pyruvate were purchased from ThermoFisher Scientific, Waltham, MA, USA. Anti-Rabbit IgG (H+L), F(ab) fragment antibody produced in goat, *N*-(3-Dimethylaminopropyl)-*N*′-ethylcarbodiimide, Tris-HCl, NaCl, Driselase, MES, Poly(ethylene glycol) 3350 MW, Sodium Azide, Tris(4,7-diphenyl-1,10-phenanthroline) ruthenium (II) dichloride, skim-milk powder, Tween-20, and Triton x-100 were purchased from Millipore Sigma, St. Louis, MO, USA. GST-tag antibody [HRP] produced in rabbit was purchased from GenScript, Piscataway, NJ, USA. Nitrocellulose membrane and Western blot chemiluminescent substrate kit were purchased from Fisher Scientific, Hampton, NH, USA. Paraformaldehyde (PFA) was purchased from Electron Microscopy Sciences, Hatfield, PA, USA. Murisage and Skoog mixture was purchased from Caisson, Smithfield, UT, USA. DYKDDDDK Tag Recombinant Rabbit monoclonal antibody was purchased from Invitrogen, Waltham, MA, USA. All commercial materials were used as received.

**Synthesis of Near Infrared Emitting Dyes**—The synthesis and characterization of red emitting chlorins P660 [33], P665 [34], P640 [35], and P690 [36] as well as near-IR emitting bacteriochlorins P710 [37,38], and P820 [39] were reported previously. The notation PXXX stands for porphyrin (P) (chlorin and bacteriochlorins are porphyrins) and the dye’s emission peak wavelength. 4,8-Dibromobenzo[1,2-*c*;4,5-*c*′]bis[1,2,5]thiadiazole BBTD-Br_2_ was prepared via literature methods [40]. BBTD-Br_2_ was further derivatized by palladium catalyzed cross-coupling utilizing Schlenk technique. Refs. [40,41,42,43,44,45,46,47] Known compounds BBTD850 and BBTD990 were synthesized following previously published procedures [46,47]. BBTD780 was synthesized ina similar manner using a Suzuki coupling reaction of BBTD-Br_2_ with N-Boc-2-pyrroleboronic acid. A flame-dried Schlenk flask was charged with BBTD-Br_2_ (150 mg, 0.426 mmol), *N*-Boc-2-pyrroleboronic acid (449.5 mg, 2.130 mmol), aqueous 2 M K_2_CO_3_ (6 mL), and THF (30 mL). The solution was then degassed 3 times by freeze–thaw-pump cycles. The flask was then charged with Pd(PPh_3_)_4_ (133 mg, 0.115 mmol) and degassed one more time by freeze–thaw-pump cycles. The reaction mixture was stirred at 60 °C for 2 h. The reaction mixture was diluted with CH_2_Cl_2_, washed with water (3 × 50 mL) and brine, dried (Na_2_SO_4_) and concentrated. Column chromatography (silica, 4:1 CH_2_Cl_2_/hexanes) provided a royal-blue film (35.1mg, 16%). ^1^HNMR (CDCl_3_, 400 MHz): 7.61–7.56 (m, ^2^H), 6.92–6.87 (m, ^2^H), 6.49 (t, J = 3.4 Hz, ^2^H), 1.22 (s, ^18^H). The dyes were characterized by ^1^HNMR using a Bruker 500 MHz Avance III HD with Cryoplatform NMR and processed by MestReNova software, Mestrelab Research, Santiago de Compostela, Spain. HRMS (MALDI-TOF) spectra were obtained with an AxION TOF, PerkinElmer, Waltham, MA, USA. Absorbance and fluorescence spectra were collected in dichloromethane on a DU 800 Spectrometer, Beckman Coulter, Pasadena, USA, and a Horiba Quantamaster 400 fluorimeter, Kyoto, Japan.

**Pdots Synthesis and Characterization**—Near infrared emitting dye-doped Pdots were synthesized via a nanoprecipitation method [11]. PFBT, PSMA, and the near infrared emitting dye were dissolved in anhydrous THF overnight, each in separate 25 mL round bottom flasks. Solutions were stored under nitrogen at room temperature, with the dyes stored away from light. The three precursors were filtered through a 0.22 μm PTFE syringe filter, then mixed together in 1 mL THF. The concentrations of each of the precursors in the mixture were 100 ppm (PFBT), 100 ppm (PSMA), 2–20 ppm (dye) depending on the dye. The solution was then injected into 10 mL DI water under vigorous sonication at room temperature for 1 min. THF was removed through vacuum evaporation on a rotary evaporator at 60 °C. All samples were filtered through a 0.22 μm cellulose acetate syringe filter prior to further studies. Oxygen sensing Pdots were synthesized with the same method, replacing PFBT with PFO and porphyrins with Tris(4,7-diphenyl-1,10-phenanthroline) ruthenium (II) dichloride. Tris(4,7-diphenyl-1,10-phenanthroline) ruthenium (II) dichloride was not soluble in pure THF but was soluble in a 10% ethanol/THF mixture. Additionally, despite the dye being partially water soluble, the dye molecules were encapsulated in the Pdots with high efficiency and the Pdots showed no measurable dye leakage in aqueous buffers. The size and morphology of the synthesized Pdots were measured using a field electron and ion (FEI) Morgagni 268 100 kV TEM, Morgangi, Hillsboro, OR, USA. Samples were prepared by placing a drop of Pdot solution on a Ted Pella copper supported grid (Ted Pella, Redding, CA, USA) and drying at room temperature. Hydrodynamic size and surface zeta potential were measured with a DTS1070 folded capillary cell in a Malvern Zeitasizer Nano (Model No. ZEN3600) instrument, Malvern, UK. UV-Vis absorption spectra of the Pdots in aqueous solution were measured with a Cary UV-Vis multicellular Peltier spectrophotometer (Model No. G9864A), Agilent, Santa Clara, CA, USA. Fluorescence spectra of the dye-doped Pdots were measured using Horiba Quantamasrter 400 fluorimeter, Kyoto, Japan. All samples were measured in DI water and excited at 450 nm. Corrected spectra were collected from 475 to 850 nm. Fluorescence quantum yields were measured in air-equilibrated solvents using tetraphenylporphyrin (TPP) in air-equilibrated toluene (Φ_f_ = 0.070)^DH^ as a standard

**Cryo-TEM analyses of Pdots**—Pdot samples were concentrated using a 3 kDa MWCO Amicon spin filters. Three microliter of the sample was loaded on glow discharged holey carbon grids (Quantifoil, Q1.2/1.3R, 300 mesh) and excess liquid was blotted away. Grids were plunge frozen in liquid ethane using a Leica EMGP2 and stored in liquid nitrogen till further use. Grids were loaded on a 300 keV Krios G3i cryo-TEM, ThermoFisher Scientific, Eindhoven, NL. Micrographs were collected using the standard EPU software along with K3 direct electron detector and a bio-quantum energy filter (Gatan, Pleasanton, CA, USA), with 20 eV slit: at a nominal magnification of 130,000X and pixel size of 0.6795 Å/pixel. Further image processing, dimension estimation and visualization was performed using ImageJ [41].

**Conjugation of secondary antibodies to Pdots**—IgG secondary antibody conjugation was achieved through a previously reported method [17]. A 1 mL aliquot of 100 µg/mL Pdot solution was mixed with 20 µL of 5% wt PEG solution (3350 MW) and 20 µL of 1 M HEPES buffer. To this mixture we added 10 µL of 2 mg/mL IgG secondary antibody solution and stirred the mixture on a vortex to ensure complete mixing. Finally, 20 µL of 5 mg/mL *N*-(3-Dimethylaminopropyl)-*N*′-ethylcarbodiimide were added and the mixture was placed on a rotary shaker for 2 h. Afterward, the mixture was transferred to a 100 kDa centrifugal filter unit alongside 10 µL of 10% wt Triton x-100 and centrifuged at 5000 RCF for 10 min to remove unbound antibody. Conjugation was confirmed by using dynamic light scattering and zeta potential measurements and Dot Blot. In Dot Blot experiments, Goat anti-Rabbit IgG secondary antibody (original 2 mg/mL, used as positive control), pure Pdots (used as negative control), and antibody conjugated Pdots were diluted indicated times and 2 μL of each sample was spotted onto the nitrocellulose membrane. After blocking non-specific sites by soaking in 5% skim milk powder dissolved in TBST buffer (20 mM Tris-HCl pH 7.5, 150 mM NaCl, and 0.1% Tween 20) for 0.5–1 h, the nitrocellulose membrane was incubated with primary antibody conjugated with HRP (GST-tag antibody [HRP] produced in rabbit) for 1 h at RT. Afterward, the nitrocellulose membrane was washed three times with TBST buffer (3 × 5 min) and incubated with Western blot chemiluminescent substrate for 1 min, and then was used to expose X-ray films in the dark room.

**Construction of FLAG-FLS2 transgenic plants**—The FLAG sequence-fused FLS2 genomic fragment was generated by a two-fragment polymerase chain reaction (PCR) approach, in which primers including the FLAG sequence were used in two separate PCR reactions. Each fragment of FLS2 was amplified by PCR from genomic DNA of wild-type plants using gene specific primers containing the FLAG sequence. An overlapping PCR reaction was then performed to generate the whole FLS2 genomic fragment with the FLAG sequence inserted behind the signal peptide sequence of FLS2. The PCR products were cloned into pDONR-Zeo (Invitrogen, Waltham, MA, USA)) by BP reaction and subsequently cloned into the binary vector pGWB1 (Invitrogen, Waltham, MA, USA)) by LR reaction. The construct was then transformed into *A. thaliana fls2* knockout mutant plants via the Agrobacterium strain GV3101 using the ‘floral-dip’ method [42]. The *fls2* knockout mutant was obtained from the Arabidopsis Biological Resource Center (ABRC, Ohio State University, Columbus, OH, USA). The transgenic plants were selected by germination on 35 μg/mL hygromycin-containing 1/2 MS medium. Homozygous T3 generation plants were used in this study. The functionality of the FLAG tagged-FLS2 receptor was confirmed by its ability to complement the phenotype of the *fls2* knockout mutant.

**Fluorescence imaging of antibody conjugated Pdots when targeting the FLS2 receptor in plant cells Arabidopsis thaliana**—Seeds, expressing FLAG-tagged FLS2 (FLAG-FLS2) at the N terminus, were grown on 0.5% phytagel with Murisage and Skoog medium for 10–14 days with photocycles of 16 h of light followed by 8 h of darkness. Plants were removed from the medium and fixed in 4% Paraformaldehyde for 1 h under vacuum before being stored in PBS at pH 7.2. Prior to immunostaining, a scalpel was used to slice the leaves to allow some chlorophyll release for clearer imaging. The plant cell wall was partially dissolved by 0.2% driselase in 2 mM MES at pH = 5.7 at 37 °C for 15 min. The membrane was then permeabilized with 1% Triton X-100 in PBS for 20 min at room temperature. Plants were then soaked on blocking buffer (1% BSA, 0.1% tween, 2 mM sodium azide in PBS) at room temperature for 20 min. Primary Rabbit anti DYKDDDDK-tag (anti-FLAG) antibodies were diluted to 1:250 in blocking buffer, and plants were incubated with primary antibodies overnight at 4 °C. Plants were rinsed in blocking buffer three times for 20 min at room temperatures. Pdots conjugated to the secondary antibody (goat anti rabbit IgG) were then diluted to 30 µg/mL in blocking buffer and incubated for 3 h at room temperature. Plants were rinsed in PBS three times for 10 min at room temperature. Leaves and root hairs were removed from the plant and placed in a glass-bottom dish with 10 µL of PBS under a 10 cm glass coverslip. These were imaged using an inverted IX73 Olympus fluorescence microscope, Olympus, Center Valley, PA, USA. These were illuminated using a 440 nm solid state laser (Crystalaser, Reno, NV, USA)) at 0.1 mW. Samples were observed using a 60× magnification, water objective lens (NA 1.2, Olympus (Shinjuku City, Japan) UPlanSApo). Images were collected through a 647 nm band pass filter with 57 nm width and 710 nm band pass filter with 40 nm width. Images were captured by a Photonmax EMCCD camera (Princeton Instrument, Trenton, NJ, USA) at 0.3 s exposure time.

**Oxygen Sensitive Pdots**—Following purification, the oxygen sensing pdots were placed into a 3 mL quartz cuvette and capped with a rubber septum and the initial fluorescence spectrum at an excitation wavelength of 344 nm was collected. Then, the solution was saturated with N_2_ or O_2_ for 5 min and the fluorescence spectrum was collected. The cycle of N_2_ then O_2_ saturation was completed 5 times.

## 3. Results and Discussion

**Figure 1:**
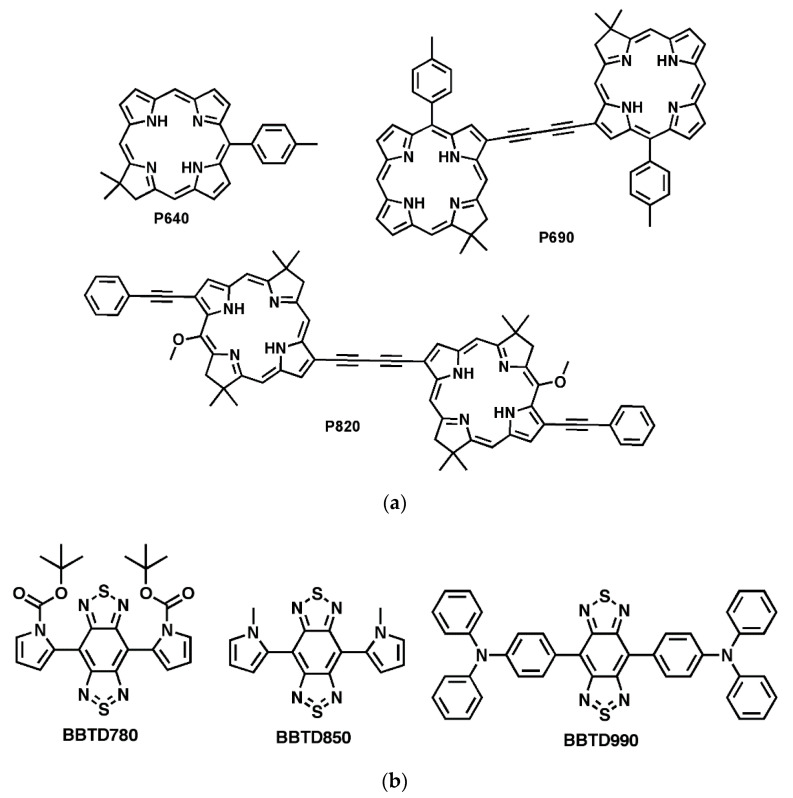
(**a**) molecular structures of porphyrin dyes used to form Pdots for near Infrared fluorescence imaging between 640 and 820 nm; (**b**) molecular structures of BBTD dyes used to form Pdots for near Infrared fluorescence imaging between 780 and 1000 nm.

**Synthesis and characterization of near infrared emitting Pdots**—Recently we reported the use of a series of hydroporphyrin fluorescent dyes as dopants to produce Pdots with tunable red-to-near infrared emission [32]. Using chlorin and bacteriochlorin as a base we used systematic structural modifications to tune the Pdots’ emission properties. The absorption and emission wavelengths can be tuned across a broad spectral range by either (a) substitution at 3,13-pyrrolic positions of the macrocyclic ring with conjugated, electron withdrawing, or electron donating substituents [38,43,44], (b) installation of an additional ring on the periphery of the macromolecule (exocyclic ring) [45], or (c) assembling of the chlorins and bacteriochlorins into strongly conjugated arrays, i.e., arrays where two macrocycles are connected by a linker which provides strong π-conjugation between subunits [39]. Their chemical structures are shown in Figure 1a. P640 and P690 are both chlorins, while P820 is a bacteriochlorin. P690 and P820 are conjugated dyads that feature the farthest red-shifted emission of their respective bases due to the extension of the conjugated π-system across the two units. Figure 1b shows the molecular structures of 4,8-Dibromobenzo[1,2-*c*;4,5-*c*′]bis[1,2,5]thiadiazole (BBTD) dyes, which are characterized with longer emission wavelengths than porphyrins. A similar nanopreciptation method was used to form BBTD-doped Pdots with an emission wavelength range between 780 and 1000 nm. The availability of a new class of BBTD dyes greatly expands the wavelength range of luminescent Pdots and enables cellular and tissue imaging in plants.

**Figure 2:**
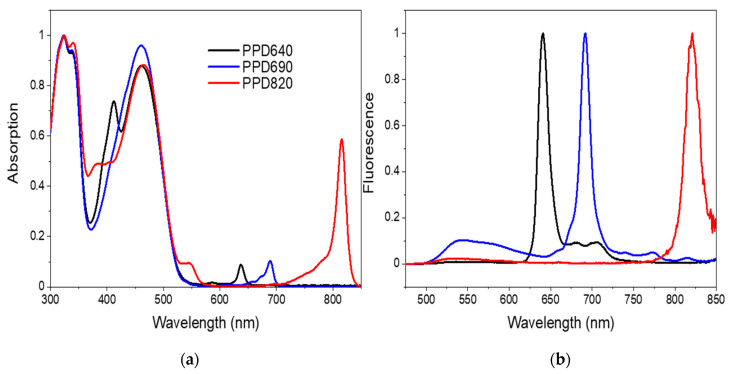
(**a**) Normalized absorption spectra of porphyrin-doped PFBT Pdots showing the main absorption peak of PFBT at 450 nm along with characteristic absorption peaks of the doped dyes at longer wavelengths; (**b**) Normalized fluorescence spectra of porphyrin-doped PFBT Pdots with emission maxima at 640 (black), 690 (blue) and 820 nm (red). The Pdots are all excited at 450 nm (PFBT peak absorption wavelength). The Pdots show strong near infrared emission while the polymer emission peak at 550 nm is nearly fully quenched.

Phorphyrin Pdots (PPD) show similar absorption spectra (Figure 2a) with a dominant PFBT absorption peak at 450 nm along with characteristic absorption peaks of the porphyrin dyes at longer wavelengths. The dye absorption peaks sufficiently overlap with the PFBT emission (data not shown). Efficient energy transfer between the polymer and dye molecules in the formed Pdots results in narrow (25 nm FWHM) fully resolved emission peaks at 640 nm (PPD640), 690 nm (PPD690) and 820 nm (PPD820) when the three types of Pdots are excited with a single excitation wavelength of 450 nm, the peak excitation wavelength of PFBT. The emission quantum yields are 0.11 for PPD640, 0.21 for PPD690 and 0.15 for PPD690. These Pdots were previously used in our laboratory in cellular imaging studies with no measurable cytotoxicity or aggregation in biological buffers and cell media [32]. The energy transfer between the polymer matrix of the Pdots and the doped dyes results in a significant increase in dye fluorescence and a corresponding decrease to near full quenching of the PFBT polymer emission at 550 nm.

**Figure 3:**
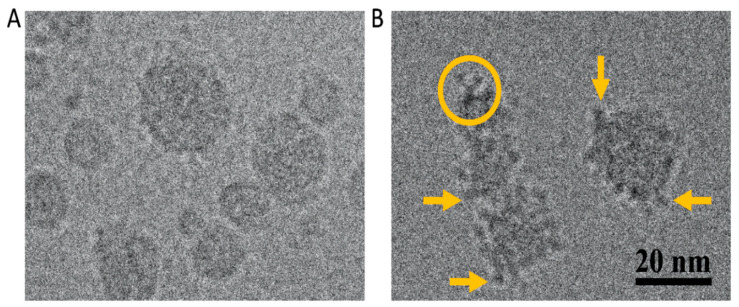
Representative Cryo-TEM images of (**A**) unmodified and (**B**) antibody-functionalized porphyrin-doped Pdots. The circle indicates a confirmed antibody, while the arrows indicate possible antibodies on the Pdots’ surface.

**Cryo-TEM of antibody coated Pdots**—Cryo-TEM measurements, shown here for the first time, reveal the Pdots to be spherical (Figure 3A) with an average diameter of 21 ± 5 nm (n = 200). This is consistent with dynamic light scattering (DLS) measurements which as expected show a slightly higher hydrodynamic diameter. Zeta potential measurements of these Pdots show them to have a negative surface charge of −35 mV, which is attributed to the presence of negatively charged carboxyl groups on the Pdots’ surface. The presence of negatively charged carboxyl groups enables the use of an EDC coupling reaction to functionalize the Pdots with goat anti-rabbit IgG secondary antibody. Figure 3B shows a Cryo-TEM image of the antibody coated Pdots. Compared to the uncoated Pdots, there are noticeable dark spots on the particles, as well as a Y-shaped of an antibody molecule. Zeta potential measurements of antibody-coated Pdots show lower negative surface charge of about −15 mV due to antibody conjugation which decrease the number of negatively charged carboxyl groups on the Pdots’ surface.

**Figure 4:**
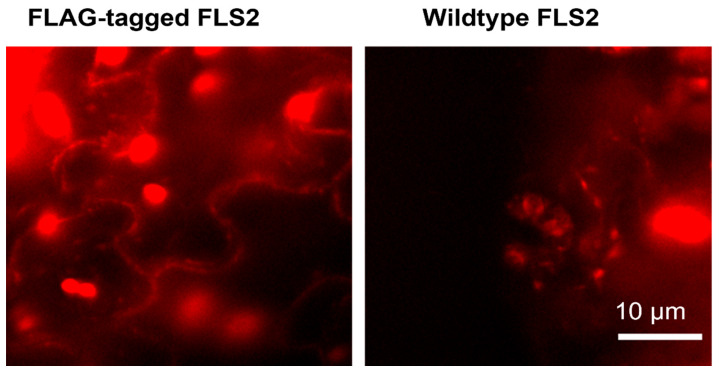
Fluorescence microscopy images of *Arabidopsis thaliana* leaf cells. FLAG-tagged FLS-2 membrane receptors are successfully labeled with antibody-conjugated Pdots (left). Wildtype leaf cells are minimally labeled with Pdots due to non-specific binding.

**Selective fluorescence imaging of leaf cells with antibody-coated Pdots**—Fluorescence images of *Arabidopsis thaliana* leaf cells tagged with antibody-conjugated 690 nm emitting porphyrin-doped Pdots are shown in Figure 4. The antibody-coated Pdots target FLS2 membrane receptors in leaf cells, which were genetically engineered to express FLAG tagged FLS2 membrane receptors. The FLAG tagged leaf cells shown in Figure 4 (left) have strong NIR fluorescence after incubation with the Pdots, while the wildtype shows very little fluorescence, indicating low non-specific binding (right).

**Figure 5:**
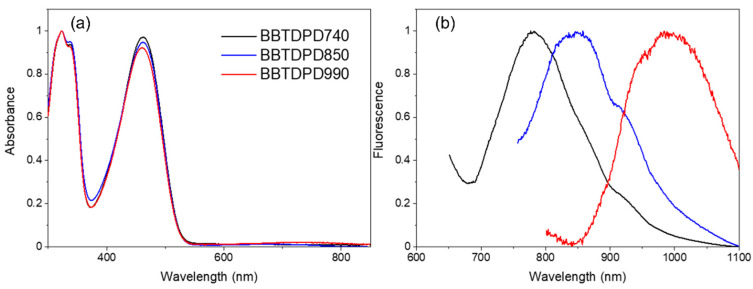
(**a**) absorption spectra of BBTD-Pdots. The spectra are dominated by the absorption of PFBT at 450 nm but lower absorption peaks of BBTD dyes at longer wavelengths between 600 and 700 nm are also seen; (**b**) fluorescence spectra of BBTD-Pdots (λex = 450 nm), demonstrating near infrared emission with emission wavelengths between 800 and 1000 nm.

**BBTD-doped Pdots**—To overcome the limitations of fluorescent porphyrins, namely their inability to reach into the NIR-II window, we investigated an alternative series of benzo-bis-thiadiazole (BBTD) dyes (see experimental section for detail). The absorbance of Pdots that are doped with BBTD dyes are shown in Figure 5a. The spectra are dominated by the absorption opeak of PFBT at 450 nm but the dyes also absorb between 620 nm to 700 nm. BBTDs utilize Donor-Acceptor-Donor (D-A-D) systems which possess an electron deficient central fluorophore covalently linked to two electron rich donors. This leads to large Stokes’ shifts and long wavelength emission [48,49]. Increasing the donating strength of the donors enables tuning BBTD emission to longer wavelengths [48,49]. Since the base BBTD has a longer emission wavelength than chlorins or bacteriochlorins, we were able to tune the emission peak wavelength of BBTD-Pdots from 700 nm all the way to 1000 nm while using a single excitation source at 450 nm (the peak excitation wavelength of PFBT). It should be noted that BBTD-Pdots show wider emission peaks than porphyrin-Pdots, which does limit their multiplexing potential. Additionally, their emission quantum yield is about 10-fold lower than the emission quantum yield of porphyrin-based Pdots, which range between 0.11 and 0.49. It is quite challenging to quantitatively measure the emission quantum yield of long wavelength emitting BBTD-Pdots due to the lack oi suitable standards and the limited sensitivity of photodetectors in this wavelength range. The quantum yield of our BBTD-Pdots is found to be around 0.01 and additional improvements in their optical properties are still needed. Nevertheless, these drawbacks are outweighed by the long emission wavelengths of BBTD-Pdots which could provide superbly high signal to noise ratio in plant cell and tissue imaging applications.

**Figure 6:**
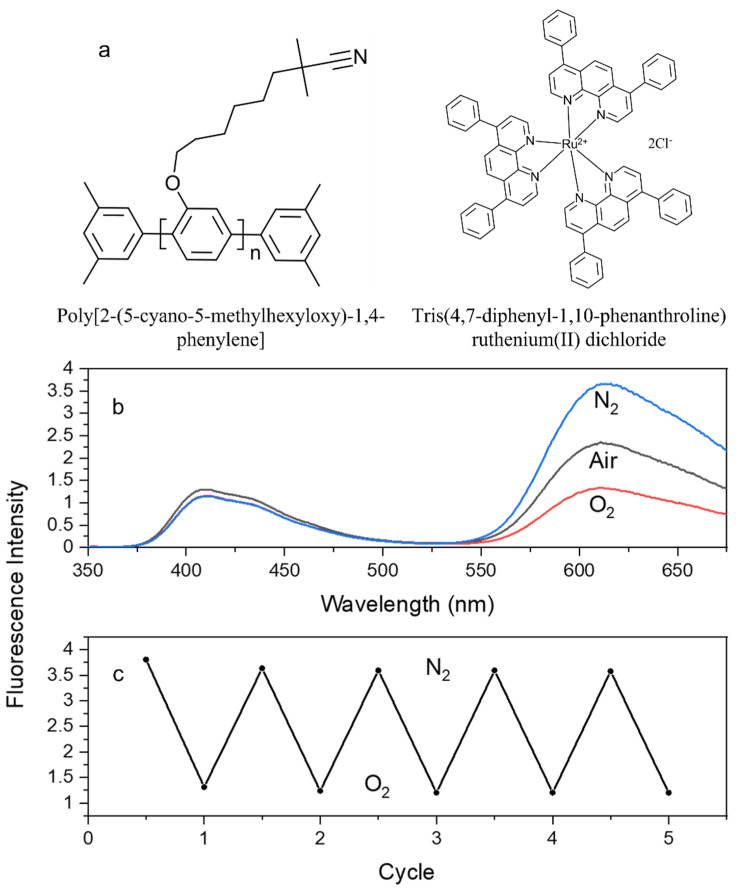
(**a**) Molecular structures of the blue-emitting polymer and Ru(dpp)_3_^2+^ used to form oxygen sensitive Pdots. (**b**) Fluorescence spectra of oxygen sensing Ru(dpp)_3_^2+^-Pdots demonstrating their oxygen sensitivity; (**c**) Demonstration of the high reversibility of Ru(dpp)_3_^2+^-Pdots when subjected to five cycles of saturating Ru(dpp)_3_^2+^-Pdots aqueous solution with nitrogen and oxygen.

**Oxygen sensitive Pdots**—While studies to expand the utility of luminescent Pdots as imaging probes are on-going, there is a growing interest in the development of Pdot-based optochemical nanosensors. Recently, we synthesized oxygen-sensing Pdot based on tris (4,7-diphenyl-1,10-phenanthroline) ruthenium (II) dichloride Ru(dpp)_3_^2+^. Ru(dpp)_3_^2+^ features a highly efficient metal to ligand charge transfer (MLCT) transition which results in a long fluorescence lifetime, in the microsecond time scale, andhigh sensitivity to O_2_ [50]. It exhibits six-times the quantum yield of Ru(bpy)_3_^2+^ and is more resistant to water-induced substitution reactions [50]. In forming oxygen-sensitive Pdots, we used a blue-emitting Poly [2-(5-cyano-methylhexyloxy)-1-4-phenylene] (CNPPP) polymer for better overlap with the the absorption spectrum of Ru(dpp)_3_^2+^. The structures of the polymer and dye are shown in Figure 6a. Ru(dpp)_3_^2+^ shows significant fluorescence enhancement when doped in the Pdot compared to the dye alone. The dye emission shows a strong response to N_2_ and O_2_ saturation while the polymer emission remaines constant (Figure 6b). To test the stability of Ru(dpp)_3_^2+^-Pdots, we subjected an aqueous solution of Ru(dpp)_3_^2+^-Pdots to five cycles of alternating O_2_ and N_2_ saturation. Figure 6c shows that throughout the five cycles, fluorescence quenching by O_2_ was completely reversible, showing that the Pdots are highly stable and effectively measure the level of oxygen under these conditions.

## 4. Summary and Conclusions

Polymer dots as fluorescence probes and sensors have rapidly advanced in recent years. The key development that led to the explosion of Pdot applications came with the discovery of dye dopants. Doping Pdots with organic dyes, either through encapsulation in the polymer matrix or through polymerization, enables combining the superb optical properties of Pdots with imaging and sensing capabilities of their doped organic fluorophores. This paper builds on a previous paper we published where we described the synthesis and characterization of near infrared emitting Pdots in detail [32]. It highlights our successful efforts to expand the use of Pdots to the near infrared region, which is necessary for quantitative imaging of biological samples that are characterized with high auto fluorescence in the visible region of the electromagnetic spectrum and high light scattering like plant cells. Our research has led to significant expansion of Pdots technology through the development and use of newly synthesized porphyrin and BBTD dyes. Conjugation of antibodies to Pdots is required for selective cellular imaging. In this paper we demonstrate for the first time the use of Cryo-TEM measurements to confirm the presence of antibody molecules on the Pdots’ surface. Furthermore, our fluorescence imaging studies show that our antibody-conjugated Pdots successfully target FLAG-tagged FLS2 membrane receptors in genetically engineered leaf cells with minimal non-specific binding. Finally, this paper demonstrates the ability to form Pdots with oxygen sensing capabilities. The simplicity of forming highly emitting Pdots and the large selection of polymers and dyes available for their formation will enable us and other researchers to form Pdots for a broad range of biological applications including in deep tissue imaging.

## Data Availability

Data used to support the findings of this study including material characterization, spectra and images are stored in digital form in the authors’ laboratories and available upon request.
